# Suitability of maize crop residue fermented by *Pleurotus ostreatus* as feed for edible crickets: growth performance, micronutrient content, and iron bioavailability

**DOI:** 10.3389/fnut.2023.1157811

**Published:** 2023-07-11

**Authors:** Martin Ventura, M. Elizabeth Holland, Michael Bartlett Smith, Jacqueline M. Chaparro, Jessica Prenni, Jonathan A. Patz, Susan Paskewitz, Tiffany L. Weir, Valerie J. Stull

**Affiliations:** ^1^Department of Entomology, University of Wisconsin–Madison, Madison, WI, United States; ^2^Department of Food Science and Human Nutrition, Colorado State University, Fort Collins, CO, United States; ^3^Department of Horticulture and Landscape Architecture, Colorado State University, Fort Collins, CO, United States; ^4^Center for Sustainability and the Global Environment, University of Wisconsin–Madison, Madison, WI, United States

**Keywords:** entomophagy, crop residue, insect agriculture, *Gryllus bimaculatus*, iron, Pleurotus ostreatus, spent mushroom substrate

## Abstract

Small-scale farming of edible insects could help combat public health challenges such as protein energy malnutrition and anemia, but reliable low-cost feeds for insects are needed. In resource-limited contexts, where grains such as maize are prohibitively costly for use as insect feed, the feasibility of insect farming may depend on finding alternatives. Here, we explore the potential to modify plentiful maize crop residue with edible mushroom mycelium to generate a low-cost feed adjunct for the farmed two-spotted cricket, *Gryllus bimaculatus*. Mushroom farming, like insect agriculture, is versatile; it can yield nutritious food while increasing system circularity by utilizing lignocellulosic residues from row crops as inputs. *Pleurotus ostreatus*, is an edible basidiomycete capable of being cultivated on corn stover (*Zea mays*). Mushroom harvest results in abundant “spent” substrate, which we investigated as a candidate feed ingredient. We created six cricket feeds containing fermented *Pleurotus* substrate plus an unfermented control, measuring cricket mass, mortality, and maturation weekly to evaluate cricket growth performance impacts of both fungal fermentation duration and mushroom formation. Pasteurized corn stover was inoculated with *P. ostreatus* mycelium and fermented for 0, 2, 3, 4, or 8 weeks. Some 4 and 8-week substrates were induced to produce mushrooms through manipulations of temperature, humidity, and light conditions. Dried fermented stover (40%) was added to a 1:1 corn/soy grain mix and fed to crickets *ad libitum* for 44 days. The unfermented control group showed higher survivorship compared to several fermented diets. Control group mass yield was higher for 2 out of 6 fermented diets. Little variation in cricket iron content was observed via ICP-spectrometry across feeds, averaging 2.46 mg/100 g. To determine bioavailability, we conducted *in vitro* Caco-2 human colon epithelial cell absorption assays, showing that iron in crickets fed fruiting-induced substrates was more bioavailable than in unfruited groups. Despite more bioavailable iron in crickets reared on post-fruiting substrates, we conclude that *Pleurotus*-fermented stover is an unsuitable feed ingredient for *G. bimaculatus* due to high mortality, variability in growth responses within treatments, and low mass yield.

## 1. Introduction

Edible insects have been promoted as a means to combat global food insecurity due to their versatile, cost-effective attributes and potential to contribute to the Sustainable Development Goals (SDGs) in regions facing undernutrition and food access challenges (1, 2). Insects are rich in protein, polysaccharides, fatty acids, and micronutrients including iron and zinc (3, 4). Many offer equivalent, and even superior nutritional profiles when compared to more commonly-consumed animal protein sources (5). Crickets contain a full complement of essential amino acids, including lysine and methionine, which are often deficient in high starch diets, and have a total protein content comparable to beef (6). Insect eating is not a new practice, as insects have been a relevant part of human diets throughout history and across the globe, supplying vital minerals, lipids, and protein (7–9). The vast majority of insects consumed today are wild-harvested and freely available during certain seasons; however, habitat destruction, climate change, chemical pollution, and over-harvesting threaten the safety and availability of this local food resource (10–15).

Insect farming offers several advantages over wild harvesting, including superior food safety, reliability, and ecosystem protection (3, 16), along with the potential to generate nutritious food year-round (17–19). Relatively low startup costs, short production cycles, and high market value for some edible species also make insect farming a promising option to improve food security, particularly in parts of southern Africa (2, 20–22). Insect agriculture can help sustainably address the complex problems of food insecurity and malnutrition because insects offer high-quality animal protein without the same environmentally degrading land-use practices common with conventional livestock production (23–25). A number of development projects seeking to support a farmed insect industry have arisen throughout Southern and Eastern Africa (20). One such initiative was established in Zambia and serves as the basis for this research. This nonprofit requested support in determining lower-cost, non-grain feed ingredients for their rural cricket colonies. At the time of this study, the organization was using a 50:50 mixture of soy and maize grain, along with variable water sources, to rear edible crickets, reporting that this method was financially unsustainable.

In Zambia, developing small-scale, local insect operations would offer both economic and nutritional benefits. Zambia is a landlocked country in southern Africa, inhabited by about 19.4 million people (26), which faces high rates of undernutrition and food insecurity. Micronutrient deficiencies are problematic; an estimated 38% of pregnant women (27) and over half of the children between 6 months and 5 years are anemic (28). Potential benefits of introducing insect farming in Zambia include a supplemental source of dietary protein and iron, new revenue streams, and improved climate resilience via diversification of local cropping systems (20, 21, 23). Moreover, insects are already widely consumed and culturally celebrated as food (25, 29–31) with at least 84 distinct species of edible insect consumed across the country (32). While these insects are typically wild harvested, some frequently farmed species, such as *Gryllus bimaculatus* (De Geer) (Orthoptera: Gryllidae), known commonly as the two-spotted cricket, are found throughout much of Africa, including Zambia, and already consumed by certain groups (32, 33).

Some insects can be inexpensively reared on materials such as post-harvest crop residues, contributing to circular economies and waste reduction (34, 35). However, successful insect rearing depends greatly on the physiological requirements of specific species and the nature of feed materials provided (36). Insect growth performance is tightly correlated with feed quality, and some studies have found that crop residues, which tend to be high in indigestible complex polysaccharides and poor in protein and micronutrients, can lead to decreased growth performance (37–39). Rearing insects on lower-quality diets containing crop residues may still be advantageous in contexts where more nutrient-dense feed is cost prohibitive, particularly if minor modifications to these substrates could improve its dietary value and enhance insect growth performance and yields. Even if locally farmed insect production systems are not fully optimized, the insects produced may offer key, and otherwise scarce nutrients to local diets, benefiting consumer health (38).

One proposed approach to improving insect growth performance when reared on crop residue-based diets is to utilize fungi to modify the nutritional profile of high-fiber, low protein substrates such as straw, maize stover, or spent grain from distillation or brewing (40). Maize (*Zea mays* (L)) is an essential agricultural staple for smallholders across southern Africa. Grown for use as both a cash crop and subsistence food in Zambia, maize covers 65% of the country’s agricultural land and receives roughly 80% of government subsidies allocated to farmers (41–43). The prevalence of Zambian maize agriculture results in an estimated 4.56 million metric tons of surplus stover annually (44). Often, post-harvest stover residues in maize fields are burned to thwart pests and reduce thatch layering to enable swifter replanting cycles (45, 46). Although this practice can suppress pest pressures and allow for more immediate availability of potassium and phosphorus to plants, it also contributes to atmospheric particulate pollution and the release of greenhouse gases (47, 48). A surplus of available maize stover could serve as a low-cost feed ingredient for edible insects, although maize stover alone may be a suboptimal feed source for farmed crickets due to a high acid detergent fiber (ADF) and low protein content (37, 38, 49). Thus, for it to be a successful bulk constituent in farmed cricket feed, chemical modification is warranted.

Fungi in the class Basidiomycetes are capable of modifying plant biomass in several potentially useful ways. White-rot fungi, such as the genus *Pleurotus* commonly known as ‘oyster mushrooms,’ can enzymatically hydrolyze some long-chain polysaccharides into shorter, more digestible forms (50, 51). Additionally, when colonized with *Pleurotus* mycelium, substrates can lose up to 40% of their carbon content to fungal respiration in the form of CO_2_, reducing the ratio of nitrogen to carbon in the substrate (52). This modification of substrate C:N ratios by fungal enzymes may render these crop residues more suitable for use as a feedstock for rearing *G. bimaculatus*, which is often protein-limited when reared on diets containing of low-protein, high-lignin crop residues (53, 54). Combining mushroom and insect farming may also be beneficial in practice, yielding two high-value and nutrient-dense foods from one system. Used mushroom substrate is considered “spent” after mushroom fruiting, and excess accumulation of this byproduct is sometimes a production constraint for mushroom farmers (55). In the most efficient oyster mushroom production systems, the ratio of spent mushroom substrate to fresh mushrooms is roughly 5:1 by dry mass (56). The estimated 2017 global yield of *Pleurotus* mushrooms was 4.7 million metric tons, meaning that a minimum of five times that amount of spent substrate required management (57). Thus, utilizing spent mushroom substrate to feed insects could present a win-win opportunity for farmers. Anecdotal evidence shared with us by cricket farmers in the United States suggests that other edible species perform well when fed spent hardwood sawdust from the shiitake mushroom, *Lentinus edodes*. However, no previous research has explored the impact of fungal-modified crop residues on growth performance and nutrient content of any farmed paurometabolous insect species such as *G. bimaculatus*.

In this study, we tested the effects of *Pleurotus ostreatus* fermented maize stover feed on cricket growth and nutritional value. *Pleurotus* mushrooms are ubiquitous, nutritious, flexible, and easy to farm, making them a good test case for this model. Specifically, we analyzed fungal colonization time on maize stover, as well as the impacts of cultivation conditions used to induce mushroom formation, or ‘fruiting,’ on several parameters of cricket growth and nutritional value, including micronutrient content and iron bioavailability. We hypothesized that increased mycelial colonization time would improve the suitability of maize stover as a cricket rearing substrate. The objectives of the study were to: (1) evaluate harvestable cricket yields; (2) determine cricket growth, maturation, and mortality responses, and; (3) establish micronutrient content and iron bioavailability of crickets reared on maize stover with varying colonization times relative to uncolonized control feed. As a secondary outcome, we were also interested in whether exposure to conditions that encourage fungal fruiting influenced these parameters as co-cultivation of both crickets and mushrooms could generate a circular food system and provide additional diversity to both the diet and potential revenue streams.

## 2. Methods

### 2.1. Cricket feed formulation

Seven experimental cricket feeds were generated, each containing 40% spent mushroom substrate colonized with *P. ostreatus* mixed with 60% of a 1:1 maize and soy grain mixture by weight. The spent mushroom substrates varied by two experimentally manipulated variables: duration of substrate colonization by fungus, and exposure or nonexposure to a fixed suite of light, humidity and temperature conditions used to induce fructification, or fruiting of mushrooms. The feed treatments were 0-week (0 W; control) feed, frozen immediately after inoculation; 2-week (2 W), 3-week (3 W), 4-week (4 W), and 8-week (8 W) unfruited substrates. Additionally, two “fruited” treatments, representing both 4- (4 WF) and 8-week (8 WF) colonization durations were tested (Table 1).

**Table 1:** Experimental feed preparation: substrate fermentation period and exposure to fruiting conditions.

Treatment name	Total days fermented post-inoculation	Days in vegetative conditions	Days in fruiting conditions
0-week (control; 0 W)	0	0	0
2-week (2 W)	14	14	0
3-week (3 W)	21	21	0
4-week (4 W)	28	28	0
4-week fruited (4 WF)	28	14	14
8-week (8 W)	56	56	0
8-week fruited (8 WF)	56	42	14

#### 2.1.1. Maize stover substrate source and preparation

Organic maize stover was obtained from the University of Wisconsin-Madison Arlington Agricultural Research Station, Arlington, Wisconsin, United States. Maize stalks were harvested from fields, dried at 48.4°C for 7 days, and mechanically chopped to achieve an average particle size of 2 cm. To simulate substrate pasteurization techniques common in *Pleurotus* production, maize stover was pasteurized in a 207 L (55 US gallon) drum heated by a propane burner (56). Distilled water was added at a ratio of 2.7 L/Kg dry stover to 5 kg dry stover in a permeable nylon bag and allowed to hydrate for 12 h. Digital temperature probes were inserted into the center of the hydrated substrate mass. The pasteurizer was filled with 140 L tap water and heated to 79°C before the bag containing stover was submerged. Substrate internal temperature was raised to 75°C 3°C and held for 1.5 h after which the vessel was drained. When liquid ceased draining from substrate bag, pasteurized substrate was transferred to a sterile 94.3 L container and cooled until the internal temperature probe reached 32°C.

#### 2.1.2. Substrate inoculation and fermentation

Maize stover was inoculated by hand mixing at a rate of 5% rye grain spawn by stover wet mass with *P. ostreatus* var. “Grey Dove” batch no. 1112 (Field and Forest Products, Peshtigo WI, United States). One kg stover aliquots were packed into polypropylene mushroom growing bags (Field and Forest Products, Peshtigo, WI). Substrate bags were placed in I36-NL Percival environmental chambers (Percival Scientific, Perry, IA, United States) set to 27°C at 75% RH and a 24D/0 L photoperiod according to vendor specifications. Substrates were numbered and randomly assigned treatments. At specified time intervals, substrates designated for fruiting were transferred into an environmental chamber programmed to maintain 18°C at 90% RH on a 12D/12 L photoperiod. Every 7 days substrates were either harvested or transferred between vegetative and fruiting conditions specified by their assigned treatments. When a substrate reached its harvest date, it was sealed and stored whole at −20°C until use.

#### 2.1.3. Final processing

Thawed stover blocks were spread 2 cm deep across screens and dried for 48 h at 26°C ± 1°C to an average moisture content of 10.3 ± 1%. Dried substrates were ground in a Wiley Mill (Thomas Wiley, Philadelphia, PA) to pass through a 2 mm screen. Each treatment combined equal amounts of powdered stover from two replicate stover samples from each of three pasteurization runs, totaling six constituent substrates per experimental treatment. Combined stover powder for each treatment was added at a 2:3 ratio by mass to a base feed. Base feed was a 1:1 mix of soybean meal and cracked maize meal, which was ground to pass through a 2 mm sieve. Mixed feeds were stored at −20°C.

### 2.2. Cricket feeding trials

#### 2.2.1. Cricket rearing conditions

In this experiment, *G. bimaculatus* crickets were reared on experimental feeds for 5 weeks. Prior to starting on the experimental feeds, crickets were reared on commercially available Mazuri cricket feed for 27 days post-eclosion (Land O′ Lakes, Inc., Minneapolis, MN, United States). This feed is a nutritionally complete diet for crickets and was given through the first 27 days of life to ensure high initial body condition in crickets. Basic ongoing colony maintenance and rearing procedures were adapted from practices common in commercial cricket husbandry and are thoroughly explained in Ventura et al. (58). Crickets were reared for the first 14 days of life in an incubator which maintained 27°C at 60% RH on a 12hD/12hL photoperiod. After 14 days they were moved into the ambient room conditions of 26°C ± 1°C at 25% ± 5% relative humidity, with a 12hL:12hD photoperiod. Crickets used in this experiment were stocked 12 February 2021 at *n* = 24 per cage, a stocking density of 300 cm^3^/individual, identical to other recent feed trials (49). Cages were randomly assigned to one of seven feed treatments. Each treatment was replicated across six cages. Crickets were cared for daily, and individual weights, as well as characteristics (maturation, sex, and number surviving), were recorded once per week (Table 2). Crickets were terminated by freezing at −20°C after 5 weeks on the experimental diets at a single timepoint (+/− 10 min), not on the basis of maturity.

**Table 2:** Description of growth, development, and survivorship parameters of crickets measured under different feed conditions.

Variable name	Data collected/characters observed	Variable of interest
Maturation	Presence of developed wings	Cage-level mean maturation
Number	n crickets per colony	Survivorship over time
Mass	Mass of individual cricket	Cage-level mean of all cricket masses
Sex	Distance between cerci, morphology of distal tergite.	Mass by sex

Experimental cages were screen-topped with 7.1 L clear polyethylene totes, each with a floor of 419.64 cm^2^. Each tote was stocked with six 15×10 cm pieces of cardboard egg carton (58). Cages were placed on two, three-tiered horticultural growing racks under T-5 full spectrum fluorescent lights. The position of each cricket habitat on these racks was cycled one tier clockwise every 2 days.

#### 2.2.2. Feed and water administration

Water access was provided *ad libitum* through the wetted surface of coconut coir packed into a 60 × 15 mm petri dish. Fresh feed was administered every 2 days. Feeding rate began at 0.5 g/cage was increased each week by 1.5 g to ensure that crickets had *ad libitum* access to feed throughout the course of the experiment.

#### 2.2.3. Nutrient and mycotoxin analysis of cricket feed

Feed analyses were conducted by Dairyland Laboratories, Arcadia, WI, United States. All feeds were analyzed for moisture, crude protein, acid detergent fiber (ADF), and neutral detergent fiber (NDF) content (Table 3), as well as micronutrient composition (Table 4). The presence of mycotoxins common to the Upper Midwest USA in the feeds were also measured.

**Table 3:** Macronutrient composition of experimental cricket feeds.

	Moisture %	Dry matter %	Crude protein %	Crude fiber %	ADF* %	Sugars %
Maize/soy base	5.94	94.06	30.08	2.13	3.07	10.98
0 W Feed	8.3	91.67	21.73	13.53	18.1	7.84
2 W Feed	7.59	92.41	20.86	14.07	20.94	9.6
3 W Feed	8.1	91.9	21.6	13.93	19.7	9.49
4 W Feed	7.73	92.27	21.24	13.76	20.74	9.63
4 WF Feed	7.57	92.43	21.53	13.09	19.92	9.43
8 W Feed	6.97	93.03	20.87	12.47	21.41	10.75
8 WF Feed	7.78	92.22	20.28	12.9	21.16	9.52

Macronutrient analysis of dry feed was conducted by Dairyland Labs, Acadia, WI in April 2021 by a single assessment per feed. *Acid Detergent Fiber (ADF) is the fiber fraction containing lignin and cellulose and is least digestible by livestock. All feeds contained 40% corn stover and 60% of a 1:1 maize:soy blend.

**Table 4:** Micronutrient composition of experimental cricket feeds based on forage analysis.

Treatment	0 W (Control)	2 W	3 W	4 W	4 WF	8 W	8 WF
Calcium (%DM)	0.26	0.27	0.26	0.29	0.29	0.34	0.33
Phosphorus (%DM)	0.37	0.4	0.39	0.42	0.39	0.4	0.39
Magnesium (%DM)	0.21	0.22	0.22	0.23	0.23	0.21	0.23
Potassium (%DM)	1.05	1.07	1.11	1.07	1.02	1.05	0.97
Sulfur (%DM)	0.17	0.17	0.17	0.16	0.18	0.17	0.17
Sodium (%DM)	0.03	0.03	0.03	0.04	0.03	0.04	0.04
Zinc (PPM)	75	71	58	63	77	66	57
Iron (PPM)	122	189	121	247	131	131	162
Manganese (PPM)	31	31	31	29	42	34	37
Copper	14	11	10	10	11	18	12
Boron (PPM)	26	17	16	17	16	17	16
Aluminum (PPM)	27	59	74	46	86	44	62

Micronutrient analysis of dry feed was conducted by Dairyland Labs, Acadia, WI in April 2021 by a single assessment per feed.

### 2.3. Micronutrient content and bioavailability of iron in crickets

#### 2.3.1. Simulated gastric digestion

Whole, freeze-dried crickets were shipped frozen from University of Wisconsin-Madison to Colorado State University and stored frozen upon arrival. Crickets were milled to a fine powder using a coffee grinder, and the following gastric digestion protocol was applied, adapted from Latunde-Dada et al. (59). One gram of lyophilized, ground crickets was combined with 10 mL of isotonic saline solution (140 nM NaCl and 5 mM KCl) and adjusted to a pH of 2.0 with HCl (1 M). Next, 0.5 mL of 16 mg/mL pepsin was added, and samples were incubated for 75 min at 37°C. The solution was raised to pH 5.5 using NaHCO_3_ (1 M) to terminate peptic digestion. Finally, 2.5 mL of a bile-pancreatin extract was added raising the pH to 7.0 with NaHCO_3_ (1 M). Isotonic solution was used to bring the digest to 16 mL and the samples were incubated for 120 min at 37°C. Once digestion was complete, samples were centrifuged at 3000 × *g* for 5 min and the supernatants were collected for further analysis.

#### 2.3.2. Quantification of iron in cricket digests and soluble micronutrient content

Total iron was measured after a simulated gastric digestion and *in vitro* mineral solubility was determined after microwave digestion followed by ICP-MS using methods of Latunde-Dada et al. (59). A Titan MPS microwave sample preparation system was used to mineralize metals present in the crickets prior to ICP-MS analysis. Two milliliters of cricket gastric digests were combined with 8 mL of nitric acid. For soluble mineral measurements, 300 mg of undigested ground cricket was added to 10 mL of nitric acid. Reactions were incubated for 15 min, then placed in the microwave digestion system for 1 h. Microwave digested samples were then diluted and analyzed by ICP-MS to determine total micronutrient profiles (whole cricket) or iron (cricket gastric digests). Elemental iron concentration was measured using an NexION 350D mass spectrometer (PerkinElmer, Branford, CT) connected to a PFA-ST (Elemental Scientific, Omaha, Nebraska) nebulizer and a peltier controlled (PC3x, Elemental Scientific) quartz cyclonic spray chamber (Elemental Scientific) set to 4°C. Calibration was confirmed by a 7-point curve prepared by serial dilution of commercially available single element standard stock solutions (Inorganic Ventures).

#### 2.3.3. Quantification of bioavailable iron in crickets

Bioavailable iron in the crickets was determined by use of a well-established Caco-2 cell assay from Sandberg (60). The human colonic cancer cell line, Caco-2, was purchased from the American Type Culture Collection. Cells were grown in tissue culture treated flasks and kept in an incubator at 37°C and 5% CO_2_ in Dulbecco’s modified Eagle’s medium supplemented with 10% fetal bovine serum, 1% minimum essential media, and 1% penicillin. Cricket digest volumes were adjusted so that each treatment received ~300 nM of soluble iron. Cells were incubated for 2 h at 37° C and 5% CO_2_ and supplemented with an additional 0.5 mL minimal media. Cells were then incubated for 22 h, after which they were washed with PBS, lysed, centrifuged for 5 min at 16,000 × *g*, and the supernatant was collected. A Human Ferritin ELISA Kit (Sigma-Aldrich, Saint Louis, MO) was used to measure bioavailable iron following manufacturer’s instructions. Final concentrations were normalized to total protein to account for variation in Caco-2 cell number.

### 2.4. Statistical analyses

Statistical analyses for cricket rearing studies were conducted in JMP PRO statistical software version 15 (SAS Institute Inc., Cary, NC). All nutrient analysis (micronutrient content and iron bioavailability) data were analyzed using Prism GraphPad version 8. Outliers were identified using the ROUT method with *Q* = 1% aggressiveness and removed prior to further analyses. Treatment differences between feeds were analyzed by one-way ANOVA at α = 0.05 with Tukey’s HSD *post-hoc* pending significant ANOVA results. Welch’s *t*-test was used for pairwise comparisons between fruited and unfruited treatments of the same duration for cricket growth parameters (weight and maturation to adult). Least-squares modeling was used to assess impacts of individual macronutrients on cricket survivorship. A standard one-way ANOVA with *post-hoc* Dunnett’s test was used to compare micronutrient content and iron bioavailability of the control group (0 W) to each experimental feed group (2 W, 3 W, 4 W, 8 W, 4 WF, 8 WF). To examine iron bioavailability in crickets reared on feeds that were or were not exposed to fruiting conditions, a two-way ANOVA was used to explore Time x Fruiting interactions. All means, unless otherwise stated, are averages for six replicate cages ± standard deviation.

## 3. Results

### 3.1. Cricket growth performance and maturation

The highest-performing feed across all response variables was the 0 W control treatment, which contained maize stover that was immediately frozen after inoculation with *Pleurotus* spawn. This control treatment yielded the highest total harvestable mass (*p* < 0.001) and showed higher individual cricket mass at harvest when compared to all other treatments (*p* < 0.001; Supplementary Table 1). Except for the comparison between 0 W (control) and 2 W feeds (*p* < 0.01), maturation rates did not differ significantly between control (0 W) and experimental feeds. Among unfruited experimental treatments, 4 W performed best, resulting in 19% greater total harvestable mass than the lowest performing unfruited fermented feed (2 W; *p* = 0.037; Supplementary Table 1). Fruiting induction had moderate negative effects on cricket maturation yield in pairwise comparison between 8 W and 8 WF feeds (*p* = 0.025; Supplementary Table 2).

#### 3.1.1. Cricket maturation

Cricket maturation was assessed weekly throughout the experiment. On average, between 53 and 80% of the crickets alive at experiment end had reached adulthood across feeds (Supplementary Table 2). Experimental week 4 was the timepoint at which most significant differences in maturation between treatments were observed and is thus the point at which we can best compare maturation effects arising from feed treatments. At this timepoint, crickets given control (0 W) feed showed a 27% higher ratio of adults to juveniles than the 2 W feed treatment (*F*_6,35_ = 3.107, *p* = 0.015).

Percent maturation was not statistically different between 4 WF and any unfruited treatments at any point during the experiment. The variance of the 8 WF group was consistently higher across experiment weeks than the 8 W feed treatment, with Levene’s test of homogeneity showing differences between the two variances at week 4 (*p* = 0.008). Due to dissimilarity in variances, the more conservative unequal variance *t*-test was used to conduct pairwise comparisons of maturation means between fruited and unfruited feeds, which showed no significant differences between unfruited and fruited substrates from both 4-week and 8-week fermentation lengths.

**Figure 1:**
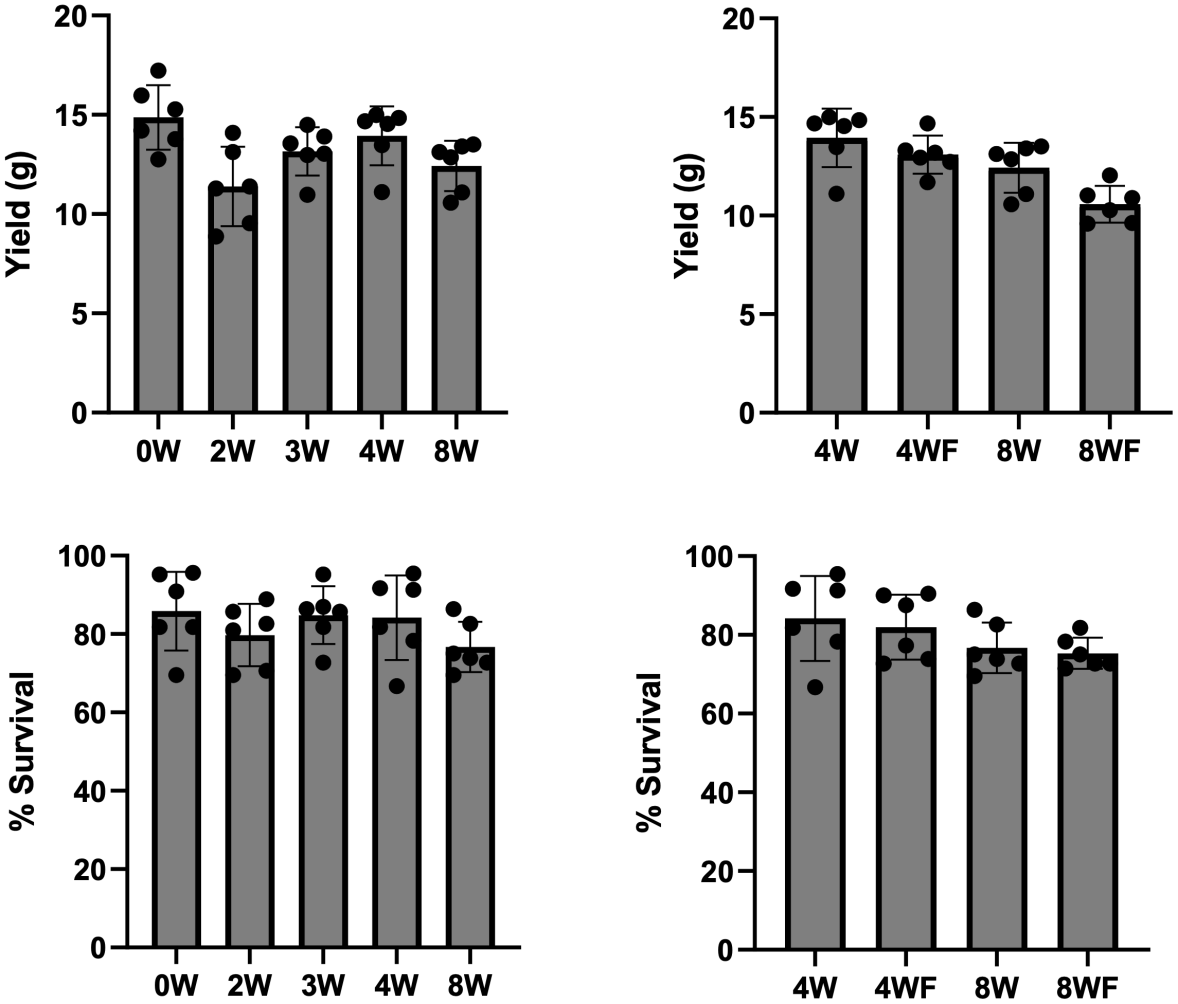
Cricket yield and survivorship by feed type. Clockwise from top left: (1) Per-cage cricket mass yield from all unfruited treatments, (2) Per-cage cricket mass yield from fruited and unfruited 4 and 8-week treatments, (3) Percent cricket survival per cage from all unfruited treatments, (4) Percent cricket survival per cage between fruited and unfruited 4 and 8-week treatments. A significant difference in yield was observed between 0 W and 2 W substrates with *F*_4,25_ = 4.529, *p* = 0.00687. A Tukey’s HSD comparing treatments 1 and 2 showed significance *Q* = 5.52 (*p* = 0.00522).

Significant differences in mean maturation were observed at the experimental week four timestep in a comparison of all unfruited substrates (Supplementary Table 3). The highest-performing unfruited treatments (0 W and 4 W) both contained significantly more adults at experimental week 4 than the slow-maturing 2 W feed (*F*_4,25_ = 4.584, *p* = 0.007, *Q* = 2.936; Figure 1). By the fifth week of observation, all treatments showed at least 90% maturation, except for the 2 W feed treatment, which had a mean maturation of 86%, a difference which was not significant when compared pairwise to the mean maturation of all unfruited treatments (*F*_4,25_ = 1.375, *p* = 0.271).

#### 3.1.2. Mortality

Mean mortality by treatment over the 5 weeks of the experiment ranged from 19.5 ± 7% to 31.6 ± 6%, with an experiment-wide average mortality rate of 24.6 ± 8%. Between the start and end of the experiment, crickets fed 2 W and 8 WF fermented stover substrates saw highest mortality of all treatments, showing 30.8% and 31.6 ± 6% mortality, respectively, over the course of the study.

At trial end, 1-way ANOVA showed significant differences in the number of surviving crickets between all feed treatments (*F*_6,35_ = 2.433, *p* = 0.045), which demonstrated feed-based differences in mean mortality, though *post-hoc* Tukey’s HSD, (*Q* = 3.125) did not show significant differences between any compared means. The responses observed in survivorship data mirror trends observed in other response variables such as mass and maturity. Crickets assigned to the 0 W and 4 W feeds had the lowest overall average mortality rates, totaling 20.4 ± 10% and 18.75 ± 8%, respectively, across the 5 weeks of the experiment.

#### 3.1.3. Total harvestable mass and mean adult mass at harvest

Total harvestable mass is a key metric that effectively combines both survivorship and mass gain. It is of primary concern to cricket farmers. Comparing all feeds, the 0 W and 4 W treatments, respectively, yielded higher average total cricket biomass than either 2 W treatments or 8 WF treatments (*F*_6,35_ = 6.374, *p* < 0.001, *Q* = 3.126) (Supplementary Table 1; Figure 1). In comparing total yield between fruited and unfruited groups, fruiting induction had a significant impact on total harvested mass with 8 W, yielding 14.8% more cricket mass than 8 WF counterparts (*t*_9.222_ = −2.875, *p* = 0.009).

*Gryllus bimaculatus* is sexually dimorphic, with observed differences in body size. The experiment-wide sex ratio for the final week of the study was 50.78% males and 49.22% females. It did not vary significantly between treatments (*F*_6,35_ = 1.146, *p* = 0.375). Female mean mass at harvest was 0.830 g ± 0.169 g, while male mean mass was 0.639 g ± 0.120 g. Female mass varied less by feed treatment, and the only significant differences detected were that females in the 0 W control and 8 W treatments showed higher mass than 8 WF crickets (*F*_6,335_, *p* = 0.015, *Q* = 2.966). Comparisons of individual mass, both combined and by sex between colonies, were similar to trends for overall yield: differences in average cricket body size were observed between 0 W control (0.78 ± 0.16 g) and 8 WF (0.615 ± 0.17 g) treatments (*F*_6,690_ = 4.313, *p* < 0.001, *Q* = 2.597), with lesser differences between the 8 WF treatment when compared to either 4 W (0.744 ± 0.012 g, *p* = 0.039), or 4 WF (0.727 ± 0.149 g, *p* = 0.036).

Variation in individual mass was higher in male crickets, and males from 0 W treatments were observed to be significantly larger than those fed 3 W, 4 W, and 8 WF feeds. Comparisons of individual mass both combined and by sex between colonies followed trends observed regarding total biomass yield, in which the most significant differences in average cricket body size were observed between 0 W control and both 8 W and 8 WF treatments (*F*_6,690_ = 4.313, *p* < 0.001, *Q* = 2.597).

Comparisons of individual mass both combined and separated by sex between colonies mirrored trends for combined-sex body mass, in which the most significant differences in average cricket body size were observed between 0 W control and 8 W fruited treatments (*F*_6,690_ = 4.313, *p* < 0.001, *Q* = 2.597).

#### 3.1.4. Feed composition

Cricket diets showed some variation in the amount of moisture, crude protein, fiber, and sugar content (Table 3). Our feeds ranged between 20.28 and 21.73% crude protein, slightly lower than the 23.18% crude protein content of the standard laboratory diet administered for the first 27 days crickets were alive. Moisture was higher, while sugar and acid detergent fiber (ADF%) concentration were lowest in 0-week diet. Single linear regression for all feeds showed no relationship between crude fiber and ADF. However, regression between total weeks fermented and ADF showed modest but significant increases in ADF with increasing duration (*F*_1,5_ = 7.925, *p* = 0.037, *R*^2^ = 0.613). Protein levels in all feeds also showed significant negative relationship with ADF, suggesting that with increasing fermentation duration, the available protein in feed decreased in proportion to cellulose and lignin (*F*_1,5_ = 9.581, *p* = 0.027, *R*^2^ = 0.657). Protein content in feeds was not significantly correlated with crude fiber but showed borderline significance when compared to total fermentation time (*F*_1,5_ = 5.541, *p* = 0.058, *R*^2^ = 0.543).

#### 3.1.5. Presence of mycotoxins

We tested experimental feeds for a suite of mycotoxins common to grain production in the Upper Midwest. The mycotoxin citrinin, a toxin produced by *Aspergillus* and other common molds (61) was detected in all fermented samples (Supplementary Table 4). The best-performing least-squares model for 5-week survival by replicate contained the predictors: ‘citrinin,’ ‘protein’ ‘crude fiber,’ ‘sugars,’ and interaction term: ‘(crude fiber*sugar)’ (*F*_5,36_ = 3.496 *p* = 0.0255, *R^2^* = 0.289). Crude protein was found to be the most significant factor impacting survivorship within this model (*F*_1,1_ = 11.079, *p* = 0.002), while citrinin also showed importance: (*F*_1,1_ = 4.9059, *p* = 0.033). These model terms were evaluated due to the previously reported impacts of protein, fiber, and carbohydrate ratios in feed on cricket mortality (37). Citrinin content (PPB) was added to these analyses due to its weak linear relationship with total fermentation time (*F*_1,5_ = 4.2282, *p* = 0.095, *R*^2^ = 0.458).

Equation:


$$\begin{array}{l} n=-61.1151+\left(0.00329\cdot c\right)+\left(3.4331\cdot p\right)+\left(0.4681\cdot f\right)+\\ \left(-0.1408\cdot S\right)+\left(f-13.3929\right)\cdot \left(\left(s-9.4967\right)\cdot -1.3318\right) \end{array}$$

Where *n* is number of live crickets remaining per colony, *c* is citrinin content, *p* is crude protein content, *f* is crude fiber, and *s* is sugar.

### 3.2. Cricket nutrient content and bioavailability

#### 3.2.1. Cricket nutrient content

In this study, the average total iron content in *G. bimaculatus* crickets across all six experimental feed treatments and the unfermented control was 2.46 mg/100 g by dry weight (Table 5), comparable to other reports for this and other farmable species (Table 6). However, the published range of iron concentrations in edible crickets is quite variable, with values as low as 1.75 mg/100 g and as high as 12.91 mg/100 g reported (Table 6). In addition, average values for calcium, zinc, and magnesium in the powdered cricket were generally less than previously reported (Table 6). There were no significant differences in total iron content of crickets among the different feed treatments. Treatment 4 W had significantly higher calcium than 4 WF, but there were also no significant differences across feed treatments in zinc or magnesium. Average crude protein content of raw crickets ranged between 25.2 and 28.0% and was not significantly different across feed treatments.

**Table 5:** Average micronutrient and protein content in crickets reared on experimental feedstocks.

Feed treatment	Fe (mg/100 g)		Ca (mg/100 g)		Zn (mg/100 g)		Mg (mg/ 100 g)		Crude protein (%) fresh weight	
0 W (Control)	2.63	+/−0.51	56.53	+/−11.61	6.45	+/−0.88	34.37	+/−2.33	26.5	+/−0.95
2 W	2.51	+/−0.35	49.82	+/−9.78	6.41	+/−1.19	38.55	+/−6.97	28.0	+/−0.40
3 W	2.33	+/−0.34	44.50	+/−7.05	5.70	+/−1.16	33.93	+/−5.85	25.2	+/−0.38
4 W	2.94	+/−0.24	59.05	+/−7.10*	6.90	+/−0.83	36.58	+/−5.92	26.2	+/−1.86
4 WF	2.16	+/−0.30	37.33	+/−3.96*	5.55	+/−0.80	32.93	+/−1.25	26.9	+/−0.30
8 W	2.52	+/−0.26	49.10	+/−5.93	7.25	+/−1.55	34.77	+/−4.40	26.9	+/−1.64
8 WF	2.23	+/−0.36	40.80	+/−11.93	6.22	+/−1.86	37.33	+/−3.28	27.2	+/−1.07

Table shows mean micronutrient and protein content in experimental crickets +/− the standard deviation across six replicates. Average micronutrient values presented as dry weight. Crude protein analyzed in triplicate on fresh weight crickets at Eurofins commercial laboratory in Madison, WI using the Kjeldahl method. No significant differences in crude protein content were detected across feed treatments based one one-way ANOVA. *Indicates mean values significantly different based on one-way ANOVA with Tukey’s *post-hoc* comparison (*p* < 0.05).

**Table 6:** Reported cricket micronutrient content across species and methods.

References	Cricket species	Iron (mg/100 g)	Calcium (mg/100 g)	Zinc (mg/100 g)	Magnesium (mg/100 g)	Method used
Ghosh et al. (6)	*Gryllus bimaculatus*	9.66	240.17	22.43	143.65	ICP-MS, dried crickets
Udomsil et al. (62)	*Gryllus bimaculatus*	7.16	105.14	14.39	72.94	ICP-MS, dried crickets
	*Acheta domesticus*	8.83	149.75	19.61	136.58	
Latunde-Dada et al. (59)	*Gryllus bimaculatus*	12.91	155.82	32.11	91.74	ICP-MS, dry cricket
		~5				ICP-MS, gastric digests of dry cricket
Kuntadi et al. (63)	*Gryllus sp.*	3.25	25.49	N/A	N/A	Not specified
Finke (64)	*Acheta domesticus*	1.75	36.6	5.43	19.3	Not specified
Payne et al. (5)	*Acheta domesticus*	5.46	104	N/A	N/A	FAO INFOODS database
Current Study	*Gryllus bimaculatus*	2.46	47.48	6.32	35.50	ICP-MS, dried cricket
		1.20				ICP-MS, gastric digests of dried cricket

Table shows comparison across studies that reported findings on average micronutrients in crickets. Species, rearing conditions, and chemical analysis methods vary between studies. All reports were converted to mg of nutrient/100 g dried cricket to standardize across studies.

#### 3.2.2. Cricket feed conversion of Iron

Large differences in iron content were observed across the various feeds given to the crickets, ranging from 121 to 247 ppm (Table 4). Interestingly, the differences in feedstock iron did not translate to significant differences in the amount of iron taken up by the crickets (Table 5). The amount of total iron found in the crickets was consistent across treatments, with an average of 2.46 mg/100 g cricket.

#### 3.2.3. Iron bioavailability

**Figure 2:**
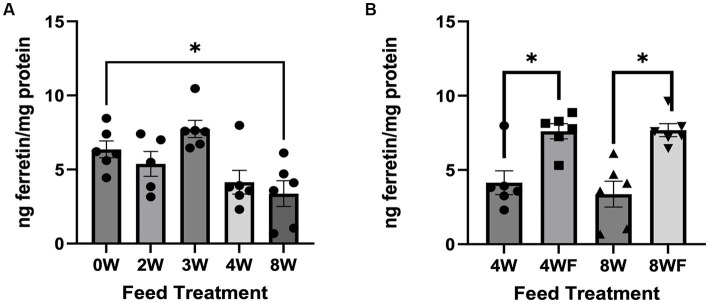
Bioavailable Ferritin in crickets reared on experimental feeds, both non-fruited treatment and fruited treatments. **(A)** Ng of ferritin per mg of protein found in each non-fruited feed treatment. **(B)** Comparison of feeds subject to fruiting conditions compared to non-fruited counterparts. Error bars represent SEM. Asterisks and connecting brackets connote differences between treatments which achieve significance below a 0.05 *P*-value threshold.

The average amount of soluble iron measured in cricket digesta was ~1.20 mg/100 grams cricket with no significant differences among the feed groups (data not shown). This data was used to normalize the total iron added to Caco-2 cells to 300 ng per well. One-way ANOVA of non-fruited treatment groups showed a significant difference among means (*F* = 5.726; *p* = 0.002). When using a Dunnett’s test to identify treatments that were significantly different from the 0 W control, only crickets on the 8 W feed treatment had significantly reduced amounts of ferritin (Figure 2A; *p* = 0.0263). Although not statistically significant, there was a general trend for reduced ferritin with longer inoculation times. Interestingly, crickets reared on feedstock subjected to 4 W or 8 W mycelial colonization times had increased bioavailable iron after exposure to fruiting conditions (4 WF and 8 WF; *F* = 27.75, *p* < 0.001 for fruiting conditions as the main effect). Pairwise comparisons showed that both the 4 W and 4 WF (*p* = 0.009) and 8 W and 8 WF (*p* = 0.001) were significantly different from one another (Figure 2B).

## 4. Discussion

### 4.1. Impact of spent mushroom substrate-based feed on crickets

Subjecting maize stover to *P. ostreatus* colonization did not produce benefits related to growth of *G. bimaculatus*. No experimental feed treatments outperformed the control within the categories of total harvestable mass, survivorship, or individual cricket mass. Cages in which crickets were fed 0 W substrates produced more harvestable cricket biomass, showed higher survivorship over the course of the study, and produced larger individual crickets than several of the fermented feed formulas. It is also clear that the negative impacts of fruiting induction on cricket survivorship led to lower harvestable yields. The effects of ‘duration’ on cricket yield are nonlinear, with the lowest-performing fermented feed durations, 2-week, and 8-week, respectively falling on the extreme ends of our fermentation time-series. Feeding *P. ostreatus* fermented stover to crickets is inadvisable in the face of our evidence, suggesting that the highest-yielding stover-containing cricket feed adjunct was the unfermented control.

#### 4.1.1. Survivorship and cannibalism

Cannibalism was observed at points throughout this experiment and is suspected to have been a principal driver of mortality within a given colony. However, the drivers are less clear. Protein deficiency is frequently implicated for cricket cannibalism in the literature, and the optimal reported dietary protein content for *G. bimaculatus* is between 22 and 30% of feed (53, 65). Experimental feeds in this study ranged from 20 to 22% crude protein by weight, indicating that protein content alone is unlikely the cause. Most crickets, including *G. bimaculatus*, will readily engage in cannibalism under general conditions of nutritional stress (65, 66). Often, mortality due to cannibalism occurs during or shortly following ecdysis, when the cricket cuticle has yet to sclerotize fully, and the cricket has a limited ability to defend against attack. Recent research on *G. bimaculatus* diets has demonstrated that crude protein may be over-emphasized as a limiting macronutrient in cricket feed and that crickets’ ability (or lack thereof) to digest complex polysaccharides present in their feed is an important driver of cricket body condition, with likely impacts on mortality (67).

We predicted that ADF, the fiber fraction least digestible to livestock, would decrease with increasing duration of fungal fermentation due to the degradation of lignin by *P. ostreatus*. Instead, linear regression between total weeks fermented and ADF showed modest but significant increases in ADF with increasing duration (*F*_1,5_ = 7.925, *p* = 0.037, *R*^2^ = 0.613). This finding comports with that of a study in which fermentation of maize stover by a related *Pleurotus* species failed to reduce lignin content in a maize stover substrate (68). Despite the importance of feed protein content in the model for survivorship, significant variations observed in cricket growth response are unlikely to arise solely from slight variation in macronutrient content between feed treatments. The relative lack of variation in feed crude protein content fails to explain much of the poor survivorship and reduced growth in crickets given some fermented feeds, leaving ample room for future investigation of the factors impacting these trends.

#### 4.1.2. Impact of fruiting conditions

The practical success of joint mushroom-insect rearing systems may depend on the impact of mushroom formation, or ‘fruiting,’ on the nutritional quality of spent mushroom substrate to be used as insect feed. This relationship has been studied to a limited degree in vertebrates and never in insects (50). *In vitro* studies using animal digestive fluids have suggested an inverse relationship between nutritional availability of a fungal substrate and the degree to which it had produced fruiting bodies (69). Work on fungal fruiting and *in vivo* ruminant responses to lignocellulosic substrates has yielded similar results, with one cattle study reporting slight decreases in ingestion rates and digestibility of fruited and unfruited wheat straw substrates fermented with *Pleurotus* (70). Evidence that fruiting leads to poorer *in vitro* digestibility and animal growth led the author of one review to contend that there is likely mutual exclusion between uses of *Pleurotus*-cultured crop residues for mushroom production and optimal end-use as animal feed (50). However, explicit studies of fungal fruiting impacts have never been conducted in insect systems. By manipulating temperature, humidity, and light, mushroom farmers encourage fungi to produce fruiting bodies at specified times, optimizing cultural controls to achieve the highest yields over the shortest period of time (56).

A key motivation for this work was to assess whether spent mushroom substrate containing principally maize stover would be an effective cricket feed adjunct once mushrooms had been harvested. We conclude, as have studies on feeding these materials to ruminants (50), that despite the insignificance of fruiting induction as a term in our linear model, the induction and harvest of mushrooms further diminishes fermented substrate value to crickets. Decreased cricket yield of insects fed the 8 WF feed compared those fed the 8 W unfruited feed, along with significantly higher mortality observed between experiment weeks 3 and 4 in 4-week fruited crickets when compared to crickets fed 4-week unfruited feed, suggests that *P. ostreatus* substrates induced to undergo fruiting efforts are a particularly unsuitable adjunct for cricket feeds. This supports our predictions that fruiting induction would lead to higher cricket mortality than unfruited substrate, though our data suggest it is unlikely that fungal allocation of nutrient resources into fruiting bodies and their subsequent depletion in spent substrate is the primary driver of these effects. Our results provide evidence that with increasing degrees of mushroom fruiting, cricket growth performance decreased, though from our data it is unanswerable whether these effects arise from effects of observed mushroom fruiting, or the cultural control manipulations we used to induce it due to these manipulations’ strong autocorrelation with resulting fruiting effort.

The inconsistent growth performance of crickets between treatment groups, which correlated poorly with macronutrient variation in feed, led us to suspect the presence of a toxicant or antinutrient. The pasteurization of fungal substrates prior to inoculation should have reduced the presence of fungal organisms capable of producing compounds toxic to crickets but left open the possibility of either residual toxicity from pre-pasteurization infection with mycotoxin-producing fungi or the establishment of cryptic fungal colonization during the feed formulation stage. While nutrients are depleted during fruiting, the process of fruiting induction also results in increased susceptibility of a substrate to infection with additional time and handling, and to the production of toxic proteins produced by *Pleurotus* for signaling and defense (71). Identifying biochemical mechanisms driving cricket growth performance falls outside the purview of this study but are likely related to the impact of mushroom fermentation duration and fruiting on the feed substrate. However, our regression modeling suggests that while presence of mycotoxins may contribute to these effects, they do not fully explain differences in cricket mortality. Additionally, the highest concentration of citrinin detected in any of our samples, found in 8 WF feed, is 500x lower than the lowest effective dose reported to modify cockroach behavior in trials evaluating mycotoxin toxicity to insects (72).

### 4.2. Relevance to human nutrition

Although we did not conduct human subjects research, this study gives insight into how cricket consumption could impact human nutrition in various contexts. For example, reliance on maize as a staple crop has contributed to several specific nutritional challenges for agrarian communities in Zambia. Maize is rich in carbohydrate content, but poor in necessary micronutrients such as iron and zinc, as well as the essential amino acid, lysine (73). Individuals who consume more than 50% of their calories from maize, such as much of the Zambian population, are at risk of protein energy malnutrition (PEM) (74). Maize also contains phytates, compounds with high affinity for chelating iron and other nutrients from consumed food and preventing absorption which can be modified via processing and cooking but may impact bioavailability of nutrients (75). The nutritional impacts of diets which rely heavily on simplified starch are particularly acute in pregnancy and early childhood, with recent reports suggesting that up to 58% of children in Zambia may experience some degree of anemia (76). The addition of nutrient-dense food to supplement maize-heavy diets is needed to improve nutrition and public health outcomes.

Our results showed that the total mineral content of crickets reared on maize stover with mushroom mycelia was generally lower than the total mineral content reported in other studies (Table 6). This could be due to differing species of cricket, different ages of crickets at harvest, analysis methods used, or what food the crickets were reared on. For example, many of the other studies reported nutrient content from crickets reared on commercial insect feeds, which may result in more nutritionally robust crickets. However, these commercial diets do not offer the same sustainability benefits as using a waste stream, such as crop residues like maize stover. Furthermore, most of the referenced studies measured total iron in ground crickets; few have evaluated the bioavailability of this iron. Only Latunde-Dada et al., referenced in Table 6, also report iron bioavailability. They compared total mineral concentrations, soluble minerals, and bioavailability of iron across grasshoppers, crickets, mealworms, buffalo worms, sirloin beef and whole wheat flour, finding that all insects had higher iron solubility than sirloin beef. Our results showed that despite differences in the iron content of the feedstocks provided to crickets (Table 4), the crickets could attain the iron they needed, with no significant differences in iron among the crickets on these feeds. This is unsurprising because iron is a tightly regulated mineral in eukaryotes (77) due to its toxicity if ingested excessively and capacity to stunt growth and development if insufficient. It should be noted that iron is not regulated through excretion, only through absorption, transport, and storage (77).

The amount of bioavailable iron was impacted by the different feed treatments. There was a significant reduction in bioavailable iron in 8 W feed compared to the 0 W control. This suggests that these active periods of mycelial growth may result in increased uptake of iron by the fungi. In fact, *P. ostreatus* is known to bioaccumulate iron in its mycelial biomass (78). More interesting still is that the insects reared on the 4 W or 8 W feeds had significantly reduced bioavailable iron compared to insects reared on 4 WF and 8 WF treatments. The fruiting conditions stimulate a change in fungal metabolism from mycelial growth to production of fruiting bodies, which may be altering fungal metabolite profiles, including secretion of chelators or other molecules that can bind iron. Iron is known to bind to different organic functional groups which can interfere with oxidation and reduction reactions of ferric and ferrous iron (79). This would in turn interfere with how the iron was absorbed by enterocytes. Identification of fungal metabolites at different stages of growth and fruiting and how they may alter iron bioavailability should be studied further.

## 5. Strengths and limitations

To our knowledge, this research is novel in several respects. The limited number of studies evaluating edible insect growth response to fermented feeds and to spent mushroom substrates have not been concerned with questions of fermentation time, a variable of strong importance to mushroom farmers. These few studies, including Li et al. (80) have tended to compare insect growth responses to feeds containing spent substrate from several different fungal species at a single timepoint: the stage when substrates have ceased to produce saleable mushroom harvests. These studies are relevant to the broader evaluation of which microorganisms might best condition lignocellulosic residues for use as insect feed, but necessarily elide the temporally linked dynamics arising from changes in the fungal organism’s metabolism and protein expression as it ferments and fruits on the substrate. Our work demonstrates that if circumstances force farmers to administer *P. ostreatus*–fermented maize stover substrate as a feed adjunct, there is likely an optimum time point during the fermentation process at which these feeds will result in minimal yield losses. Similarly, we believe our work to be the first to expressly investigate the effects of fungal fructification on cricket growth. While the limitations of our study design prevented us from demonstrating the effects of multiple mushroom harvests, we nonetheless show that substrates which have undergone fruiting show inferior potential as a feed adjunct for crickets as indicated by depressed mass and total yield.

We could identify no studies to date in which edible fungus or its spent substrate was administered to a paurometabolous insect for evaluation as feed. The handful of studies of which we are aware investigate the growth response impacts of these materials in the yellow mealworm, *Tenebrio molitor*, (Linnaeus) (Coleoptera: Tenebrionidae), the figeater beetle, *Cotinus mutabilis*, (Gory and Percheron) (Coleoptera: Scarabeidae), and the black soldier fly, *Hermetia illucens* (Linnaeus) (Diptera: Stratiomyiidae). These organisms, while members of superclass Insecta, are descendants of far more recent lineages than the paurometabolous orthopteran, *G. bimaculatus* (81, 82). Both mealworms and soldier flies possess digestion systems which allow them to detoxify or derive energy from a range of toxic proteins and mycotoxins, along with certain plastics in the case of mealworms (83–85). Our findings suggest strongly that *G. bimaculatus* may be more susceptible to the impacts of toxicants derived from fungal fermentation than the other insects in which these types of feeds have been tested. Additionally, further investigation of the spent substrate from other fungal species is warranted. Anecdotal reports from cricket farmers in the United States have suggested that *Gryllodes sigillatus* (Walker) (Orthoptera: Gryllidae) perform well when fed spent hardwood sawdust substrate from the shiitake mushroom, *Lentinus edodes*. Spent shiitake substrate was evaluated as a plausible feed ingredient in *Tenebrio molitor* (Linnaeus) and *Hermetia illucens* (Linnaeus) insect diets (86), but has yet to be assessed in cricket or other Orthopteran diets.

## 6. Conclusion

This study was conducted as a laboratory simulation of a farming scenario and was concerned primarily with impacts immediately pertinent to cricket farming success and nutrition, while leaving many intriguing entomological questions unexamined. Cricket growth results from this trial indicate that spent substrate from *P. ostreatus* grown on maize stover is a poor feed substrate for farming crickets. However, despite differences in quality and nutrient content of the feed, crickets reared in this study maintained valuable levels of bioavailable iron, similar to those reported elsewhere.

Further investigations on cricket physiology, microbiome, gene expression, and behavior must be conducted to draw credible conclusions about the underlying factors leading to variation in farmed insect growth performance on novel feed materials. *G. bimaculatus* is an increasingly popular model organism with a fully-sequenced genome and is also a mainstay of insect agriculture production systems in many parts of the world (87). This dual utility results in a slimmer knowledge gap toward conducting high-resolution sequencing work on this cricket compared to many other non-model insect species, while its widespread use as minilivestock means that new information generated through these processes will be more relevant toward informing management choices and approaches to feed formulation.

Additionally, given the unexpected superiority of the unfermented control feed (0 W), these results comparing 0 W stover to fermented cricket feeds warrant replication. The use of unfermented stover as a feed adjunct should be compared to feeds in use by cricket growers. And, given the substantial energy output required to heat water sufficiently to achieve successful *Pleurotus* inoculation of maize stover and similar plant biomass, it will be important to understand the role that pasteurization plays in the nutrient availability and growth performance of these feeds.

Last, our demonstration of the inappropriateness of a single commercial strain of one fungal species for use in conditioning cricket feed does not preclude the likelihood that other microorganisms may be able to transform crop residues into high-value feed for crickets and other insects more efficiently. There are more than 100 species of saprobic edible fungi which can be cultivated and diversity of countless subspecies and strains therein (88). We remain optimistic that the diversities of chemical ecology, substrate preference, and growth habits contained just within those fungal species which have been adapted to culture by humans offers high potential for utility as a feed conditioner for edible insects. Future work should evaluate insect growth responses to a wider range of fungal cultivars, with specific attention to the impacts of toxic secondary metabolites produced by fungi.

Regardless of the differences in bioavailable iron in crickets reared on different feedstocks, our data suggest that crickets could be a valuable source of iron in a Zambian diet. The average iron recommended daily allowance (RDA) across all ages and genders, not including infants or women who are pregnant or breastfeeding, is 12 mg per day. Based on a typical serving size of 3.5 ounces, the crickets grown on maize stover, and mushroom feedstock would provide ~20% of the recommended daily value of iron for the average person. Based on this information, the FDA would likely label crickets as an excellent source of iron. Additional research, especially animal studies and human intervention trials, is needed to confirm the nutritional quality and bioavailability of farmed edible crickets.

As in many low and middle-income African countries, rates of childhood anemia are high, with up to 58% of children under five showing signs of moderate to severe anemia (76). Modeling suggests that certain recalcitrant nutritional problems could be alleviated in part through the increased dietary consumption of animal-derived foods containing ample digestible protein, along with substantially higher content of essential micronutrients such as calcium, zinc, and available iron (89). However, cattle farming and beef consumption tend to be the purview of wealthier Zambians, and for many, eating beef is unaffordable (23, 90). Increasing insect farming, resulting in more consistent edible insect availability for consumption could help alleviate these issues. A recent model found that adding as little as 5 g/day dried edible insects to diets across regions of Africa and Asia could increase dietary iron intake and result in significant reductions in the number of people at risk of nutrient deficiency—yielding 67 million fewer people at risk of protein deficiency, 166 million fewer people at risk of zinc deficiency, and 237 million fewer people at risk of folate deficiency (91). For nutritional and environmental benefits of *G. bimaculatus* to be realized, farming methods using low-cost, available substrates that protect insect growth performance and nutrient content are still needed.

## Data availability statement

The original contributions presented in the study are included in the article/Supplementary material, further inquiries can be directed to the corresponding author.

## Author contributions

MV and MH conducted the experiments, collected, analyzed the data, and wrote the manuscript. VS conceived of the study, obtained funding, initiated collaborations, oversaw data collection, and helped wrote the manuscript. TW helped design the study, oversaw data collection, and helped wrote the manuscript. MS, JP, and JC assisted with the experiments and collected data. JAP helped obtain funding and wrote the manuscript. SP helped obtain funding, oversaw data collection, assisted with data analysis, and helped wrote the manuscript. All authors contributed to the article and approved the submitted version.

## Funding

This study was obtained through an internal Contemporary Social Problems Award at UW-Madison (VS/SP) and Colorado Agricultural Experiment Station grant COL00407 awarded to TW and VS.

## Conflict of interest

The authors declare that the research was conducted in the absence of any commercial or financial relationships that could be construed as a potential conflict of interest.

## Publisher’s note

All claims expressed in this article are solely those of the authors and do not necessarily represent those of their affiliated organizations, or those of the publisher, the editors and the reviewers. Any product that may be evaluated in this article, or claim that may be made by its manufacturer, is not guaranteed or endorsed by the publisher.
