# Supplementary material for: Suitability of maize crop residue fermented by Pleurotus ostreatus as feed for edible crickets: growth performance, micronutrient content, and iron bioavailability
Source: Front Nutr. 2023 Jul 11;10:1157811. doi: 10.3389/fnut.2023.1157811 (PMC10368478; doi:10.3389/fnut.2023.1157811)
Supplement: Supplementary file 1 [file Table_1.DOCX]

Supplementary Material

Suitability of maize crop residue fermented by *Pleurotus ostreatus* as feed for edible crickets: growth performance, micronutrient content, and iron bioavailability

Martin Ventura^1^, M. Elizabeth Holland^2^, Michael Bartlett Smith^1^, Jacqueline M. Chaparro^3^, Jessica E. Prenni^3^, Jonathan A. Patz^4^, Susan Paskewitz^1^, Tiffany L. Weir^2^, Valerie J. Stull^4^*

*** Correspondence:** vstull@wisc.edu.

# Supplementary Figures Tables

**Supplemental Table 1:** Total harvestable mass by experimental feed at harvest

| **Treatment** | **Number of Replicates** | **Mean Cage Yield (g)** |  | **SD (g)** | **Tukey HSD Connecting letters** |
| --- | --- | --- | --- | --- | --- |
| 0W (Control) | 6 | 14.8682 | +/- | 1.6183 | A* |
| 2W | 6 | 11.3888 | +/- | 2.0038 | B |
| 3W | 6 | 13.158 | +/- | 1.2112 | AB |
| 4W | 6 | 13.9387 | +/- | 1.4848 | A** |
| 4WF | 6 | 13.0893 | +/- | 0.9701 | AB |
| 8W | 6 | 12.4292 | +/- | 1.268 | AB |
| 8WF | 6 | 10.5762 | +/- | 0.9403 | B |
| Table depicts results of ANOVA comparing mean total yield per cage for all treatments at harvest (Week 5). Treatments with one or more shared connecting letter indicate that Tukey’s post-hoc test detected no significant (p<0.05) differences in mean per-cage yield for the treatments to which letters correspond. Those with a * indicate significant difference: *0W vs 2W: *P* = 0.002 *0W vs 8W fruited: *P* <0.001 **4W vs 2W: *P* = 0.037 **4W vs 8WF: *P* = 0.005 | | | | | |

**Supplemental Table 2:** Mean percentage of adult crickets per cage across all experimental treatments in the penultimate sampling

| **Treatment** | **%Adults in cage** |  | **SD (%)** |
| --- | --- | --- | --- |
| 0W (Control) | 80.3 | +/- | 5.7 |
| 2W | 53.2 | +/- | 18.9 |
| 3W | 64.6 | +/- | 14.7 |
| 4W | 75.1 | +/- | 8.6 |
| 4WF | 71.2 | +/- | 9.1 |
| 8W | 72.6 | +/- | 6.2 |
| 8WF | 59.9 | +/- | 19.4 |

All experimental feeds tested on in 6 replicates.

**Supplemental Table 3:** Mean percent of adult crickets present in cages at experiment week 4

| **Treatment** | **Number of Replicates** | **%Adults in cage** |  | **SD (%)** | **P (*T*< *t)*** |
| --- | --- | --- | --- | --- | --- |
| 4W | 6 | 75.1 | +/- | 9.16 | *P =* 0.419 |
| 4WF | 6 | 71.2 | +/- | 8.69 |  |
| 8W | 6 | 72.6 | +/- | 5.78 | *P =* 0.087 |
| 8WF | 6 | 59.95 | +/- | 19.7 |  |
| Results of Welch pairwise unequal variance t-tests comparing mean percentage of adult crickets per cage unfruited treatments at experiment week 4. | | | | | |
|  | | | | | |

**Supplemental Table 4.** Mycotoxins Present in Experimental Feeds

|  | DonsDry  (PPM) | Zearalenone  Dry (PPB) | T2/HT2  Dry (PPB) | Fumonisin  Dry (PPB) | Citrinin  Dry (PPB) |
| --- | --- | --- | --- | --- | --- |
| 0W Control | 0.53 | 202.22 | 18.03 | 0 | 0 |
| 2W | 0.15 | 0 | 8.49 | 0 | 201.24 |
| 3W | 0.11 | 0 | 0 | 0 | 190.22 |
| 4W | 0 | 0 | 0 | 0 | 721.77 |
| 4WF | 0.18 | 0 | 15.16 | 0 | 112.31 |
| 8W | 0 | 0 | 0 | 1.03 | 331.53 |
| 8WF | 0 | 0 | 0 | 0 | 985.75 |

Dry feed samples were sent to Dariyland Labs, Acadia, WI, and tested for common mycotoxins.
